# Characterization of strontium-rich groundwater in a typical alpine basin on Tibetan Plateau: Implications for sustainable exploitation and development

**DOI:** 10.1371/journal.pone.0331449

**Published:** 2025-09-10

**Authors:** Shanhu Xiao, Jie Wang, Yong Xiao, Shaokang Yang, Yuqing Zhang, Jianhui Wang, Huizhu Chen, Wenxu Hu, Liwei Wang, Guangbin Zhu, Yanting Su, Dongyang Zhao

**Affiliations:** 1 Bureau of Qinghai Environmental Geological Prospecting, Xi’ning, China; 2 Qinghai 906 Engineering Survey and Design Institute Co., Ltd., Xi’ning, China; 3 Faculty of Geosciences and Engineering, Southwest Jiaotong University, Chengdu, China; 4 Key Lab of Environmental Geology of Qinghai Province, Xi’ning, China; 5 Qinghai Engineering Research Center of Geoenvironment Protection and Geohazard Prevention, Xi’ning, China; 6 School of International Studies, Chengdu College of Arts and Sciences, Chengdu, China; 7 MOE Key Laboratory of Groundwater Circulation and Environmental Evolution, China University of Geosciences (Beijing), Beijing, P. R. China; 8 Sichuan Province Engineering Technology Research Center of Ecological Mitigation of Geohazards in Tibet Plateau Transportation Corridors, Chengdu, China; Institute of Urban Environment Chinese Academy of Sciences, CHINA

## Abstract

This study focuses on mineral groundwater in alpine regions and its sustainable exploitation. The Tongde basin on Tibetan Plateau was investigated to reveal the hydrochemistry and formation of mineral groundwater in alpine basins and its sustainable development under anthropogenic disturbances. The results show that groundwater there is characterized by enriched strontium, with concentrations in the range of 0.29–2.03 mg/L, exceeding the mineral water threshold of 0.20 mg/L. Groundwater has large salinity variation but is predominantly fresh, with an average TDS of 456.72 mg/L and a dominant hydrochemical facies of HCO_3_-Ca. Groundwater chemistry is primarily governed by water-rock interactions, particularly silicates weathering, carbonates dissolution and cation exchange. The enriched Sr in groundwater primarily originate from the dissolution of silicate minerals and carbonate cements in arkosic sandstone and argillaceous siltstone, with albite, calcite, and strontianite being the primary minerals involved. Groundwater quality in the basin is mostly excellent to good, with EWQI values below 100. While agricultural practices have introduced nitrogen contaminants (NH_4_^+^, NO_2_^-^ and NO_3_^-^) into groundwater. Among them, NO_3_^-^ would pose potential health risks to various populations and should be addressed in the development of alpine basins with the endowment of mineral water resources.

## 1. Introduction

Mineral water, a natural resource with unique health benefits, has garnered significant attention in recent years for its potential applications in health and industry [1–5]. The presence of essential trace elements such as strontium, magnesium, and calcium in mineral water has been widely recognized for their positive impacts on human health, including bone health, cardiovascular function, and overall well-being [6–9]. Among these, strontium-rich mineral water has emerged as a particularly valuable resource due to its potential therapeutic properties [4,10–12]. The study of such specialized mineral waters is crucial not only for understanding their geological origins but also for assessing their sustainable exploitation and development [13,14].

Over the past few decades, research on mineral water has made significant progress across the world [6,15–21]. Studies have shown that high-altitude regions, particularly those with complex geological structures and unique hydrological conditions, are favorable for the formation of mineral water [4,17,22]. These regions often exhibit high levels of trace elements due to enhanced rock-water interactions and natural filtration processes [15,23]. Alpine regions in various parts of the world have been identified as key areas for the occurrence of high-quality mineral water [13,15–17,24]. However, despite these advancements, there remains a gap in understanding the specific mechanisms that contribute to the formation and enrichment of strontium in groundwater, especially in high-altitude source areas.

The Tibetan Plateau, often referred to as the “Asia Water Tower” [25,26], is an ideal region for exploring the formation and characteristics of mineral water in alpine areas due to its high altitude, low temperature, and pristine environment. This plateau is home to numerous alpine basins that host a variety of mineral water resources [22,27], including those rich in strontium [4]. However, with increasing human activities such as urbanization, tourism, and resource exploitation, the Tibetan Plateau is facing growing environmental pressures [28–33]. These activities may impact the quality and sustainability of mineral water resources. Therefore, it is imperative to clarify the hydrogeochemical mechanisms that govern the formation of mineral water and to assess the suitability of these waters for sustainable development and exploitation.

To achieve a comprehensive understanding of mineral water, various techniques have been employed in recent studies [1,16,34–37]. Hydrogeochemical analysis helps identify the chemical composition and major processes influencing groundwater quality [1,38–44]. Isotopic tracing provides insights into the sources and pathways of groundwater [4,45–47]. Numerical modeling aids in simulating the dynamics of groundwater flow and solute transport [31,48], but its application in high-altitude regions like the Tibetan Plateau presents unique challenges due to the remote property and insufficient data. Thus, integrating hydrochemical and environmental isotope approaches becomes the optimal solution to unravel the formation of mineral water resources in these remote alpine regions.

This study aims to address the knowledge gaps in the characterization and sustainable exploitation of strontium-rich groundwater in a typical alpine basin on Tibetan Plateau. Specifically, the objectives of this research are (1) to investigate the hydrochemical characteristics of strontium-rich groundwater in present alpine basin, (2) to elucidate the hydrogeochemical mechanisms and sources of strontium enrichment in groundwater, and (3) to evaluate the suitability of strontium-rich groundwater for sustainable development and exploitation. By achieving these goals, this study will provide valuable insights into the sustainable management of mineral water resources in high-altitude regions and contribute to the broader understanding of groundwater dynamics in alpine environments.

## 2. Study area

**Fig 1:**
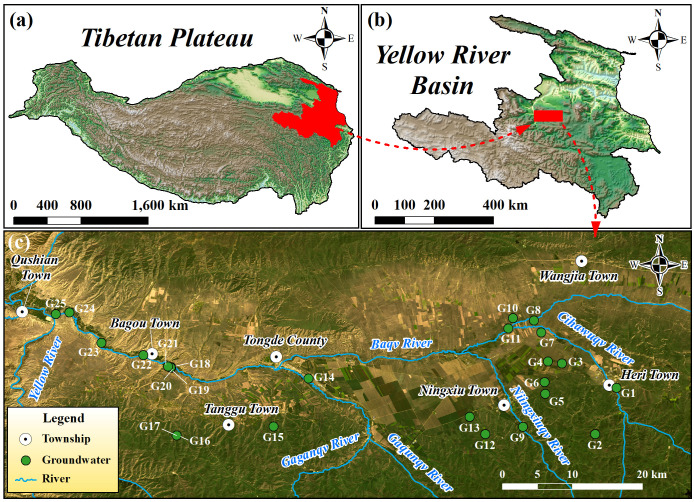
Location of (a) the upper-stream area of Yellow River watershed, (b) the Tongde Basin, and (c) the sampling sites of groundwater. (The map was created via ArcGIS 10.2 (https://www.esri.com/en-us/arcgis/products/arcgis-desktop/resources) based on the publicly available landsat 8 collection 2 level 2 data from Geospatial Data Cloud site, Computer Network Information Center, Chinese Academy of Sciences (http://www.gscloud.cn)).

The Tongde Basin is located at the overlap of the northeastern part of the Tibetan Plateau and the Yellow River watershed. It is a typical alpine basin on the Tibetan Plateau and an important source area for the Yellow River. The basin’s longitude ranges from 100°12′37″E to 101°5′33″E, and its latitude ranges from 35°5′11″N to 35°25′44″N, covering an area of 3,063 km^2^ (Fig 1). Mountains surround the basin to the north and south, while the central part is a plain. The overall terrain is oriented east-west, with higher elevations on the north and south sides and lower elevations in the middle. The eastern part is higher than the western part. The downstream area of the basin shows significant fluvial erosion and a large elevation drop. Elevations in the region vary from 2,650 m to 4,531 m. The main river in the basin is the Baqv River, which flows westward through the basin and eventually drains into the Yellow River. The climate type in the region is a continental plateau climate. Annual temperatures range from −0.77 to 1.69°C, with an average of 0.64°C. Over 80% of the annual precipitation occurs from May to September, totaling 688 mm. The annual evaporation rate is 790 mm, slightly higher than the precipitation. The climate in the basin is characterized by cold temperatures and relatively dry conditions.

The aquifer system in the study area consists of phreatic and confined water in the Quaternary loose deposits, as well as fracture water in the bedrock. The aquifer systems in the mountainous areas beyond the basin to the north and south are primarily composed of bedrock fracture water, with individual spring discharges exceeding 1 L/s. Inside the basin, the plains are characterized by phreatic and confined water in the Quaternary loose deposits. The water-richness of the aquifers decreases from upstream to downstream. At the upstream of the plains, the aquifers have good hydraulic properties, with both phreatic and confined water coexisting. Near the pediments and riverbanks, the water-richness of the aquifers increases. In areas close to the river, the yield of phreatic water from individual wells can exceed 5,000 t/d, while the yield of confined water ranges from 100 to 1,000 t/d. In the downstream plains, the hydraulic connectivity of the aquifers is weaker, with phreatic water being predominant. The yield of phreatic water from individual wells ranges from 100 to 1,000 t/d, and there is a large area of aquifer pore water being drained near the mountain front on the south side. The abundant groundwater resources and the presence of confined water upstream provide excellent material and dynamic conditions for the formation and emergence of mineral water.

## 3. Material and methods

### 3.1. Water sampling and analyses

A total of 26 phreatic groundwater samples were collected across the basin during July-August 2024. The samples in the upstream area of the basin are relatively evenly distributed, mainly along the south bank of the Baqv River and the mountains and pediments on the west bank of the Cihawuqv River. In the downstream area, most samples are distributed along the Baqv River, with a few located in the mountains on its south bank. These samples represent the condition of mineral water in the basin. During sampling, each well was pumped for over 15 minutes to eliminate the influence of standing water. All target groundwater samples were tested in situ for parameters such as pH before sampling. The samples were collected in 500 mL high-density polyethylene bottles that had been pre-rinsed 3–5 times with the target groundwater. After collection, the samples were stored at 4°C and transported to the laboratory for analysis within 48 hours.

In-situ parameter measurement (HACH HQ4D, Californian, USA) was conducted using portable measurement devices. In the laboratory, major cations (Na^+^, K^+^, Ca^2+^, Mg^2+^) and Sr were analyzed using an inductively coupled plasma mass spectrometer (Agilent 7500ce ICP – MS, Tokyo, Japan). An ion chromatography (Shimadzu LC-10ADVP, Kyoto, Japan) was employed to analyze major anions (SO_4_^2-^, Cl^-^, NO_3_^-^, NO_2_^-^) and NH_4_^+^. Acid-base titration and gravimetric analysis were used to measure HCO_3_^-^ and TDS, respectively. Sr was separated from the matrix using Sr-Spec resin and then analyzed for isotopes using thermal ionization mass spectrometry (Thermo Fisher Scientific) [49]. To ensure accuracy, each sample was tested three times with standard and blank controls interspersed. The results showed that the ionic charge balance error percentage of the groundwater samples was within ±5%, indicating reliable results.

### 3.2. Entropy-weighted water quality index

The Entropy -weighted Water Quality Index (EWQI) represents an enhancement of the traditional Water Quality Index (WQI) [50]. As the predominant water quality assessment tool, EWQI incorporates entropy to minimize human subjectivity [51]. It assigns specific weights to each factor. It serves as a reliable standard for evaluating groundwater quality [52–54]. The specific calculation formula for EWQI is shown in Eq. 1.


$$EWQI=\sum_{j=1}^{n}\omega_{j}q_{ij}$$ (1)

Where, *ω*_*j*_ is the entropy weight of the j^th^ water chemical parameter involved in water quality analysis; *q*_*ij*_ is the quality rating scale for different parameters. The calculation formulas for *ω*_*j*_ and *q*_*ij*_ are Eqs. 2 and 3, respectively.


$$\omega_{j}=\frac{1-e_{j}}{\sum {\mathrm{\backslash nolimits}}_{j=1}^{n}\left(1-e_{j}\right)}$$ (2)

$$q_{ij}=\frac{x_{ij}}{S_{j}}\times 100$$ (3)

$$X=\left[\begin{array}{cccc} {}*20cx_{11} & x_{12} & \cdots & x_{1n}\\ x_{21} & x_{22} & \cdots & x_{2n}\\ \vdots & \vdots & \ddots & \vdots \\ x_{m1} & x_{m2} & \cdots & x_{mn} \end{array}\right]$$ (4)

Where, *e*_*j*_ denotes the information entropy of the j^th^ water chemical parameter. *S*_*j*_ represents the acceptable value of the j^th^ water chemical parameter as per the Chinese drinking water standards and the World Health Organization’s recommendations. *X* is the feature matrix of each groundwater sample for each water chemical parameter. *X*_*ij*_ is the feature value of the j^th^ water chemical parameter for the i^th^ groundwater sample. The calculation formula for the information entropy of each water chemical parameter is shown in Eq. 5.


$$e_{j}=-\frac{1}{\ln m}\sum_{i=1}^{m}p_{ij}\ln\left(p_{ij}\right)$$ (5)

Where, *p*_*ij*_ is the proportion of the i^th^ groundwater sample in the j^th^ water chemical parameter. Its calculation formula is given in Eq. 6.


$$p_{ij}=\frac{y_{ij}}{\sum {\mathrm{\backslash nolimits}}_{i=1}^{m}y_{ij}}$$ (6)

Where, *y*_*ij*_ represents the value of *x*_*ij*_ after normalization. The normalization process is shown in Eq. 7.


$$y_{ij}=\frac{x_{ij}-\min\left(x_{ij}\right)}{\max\left(x_{ij}\right)-\min\left(x_{ij}\right)}$$ (7)

### 3.3. Human health risk assessment

This study employs the Human Health Risk Assessment (HHRA) model developed by the USEPA [55] to assess the potential health risks to the public from contaminated groundwater. This model provides a practical approach for ensuring safe water supply by quantitatively characterizing the health impacts of exposure to polluted groundwater [56–58]. The primary exposure pathways are oral intake and dermal contact [59]. Consequently, the study focuses on estimating health risks through these two main pathways. The population is divided into four groups: infants, children, adult females, and adult males, considering physiological differences [60]. Parameters used in the HHRA model for various populations are listed in S1 Table. The process of determining the potential health risks of pollutants to humans using the HHRA model is as follows:

Step 1: Calculate the estimated Chronic Daily Intake (*CDI*) and Dermal Absorption Dose (*DAD*) of specific pollutants. The calculation formulas are given in Eqs. 8 and 9.


$$CDI=\frac{C_{i}\times IR\times EF\times ED}{BW\times AT}$$ (8)

$$DAD=\frac{C_{i}\times K_{p}\times SA\times T\times EF\times ED\times EV\times CF}{BW\times AT}$$ (9)

Where, *C*_*i*_ denotes the concentration of each toxic ion involved in the health risk assessment. *IR* is the daily water intake. *EF* represents the exposure frequency. *ED* is the exposure duration. *BW* stands for the body weight of the assessed population. *AT* is the average time of exposure occurrence. *K*_*p*_ is the dermal permeability coefficient. *SA* represents the skin contact surface area. *T* is the contact duration. *EV* is the average frequency of skin contact. *CF* is the unit conversion factor.

Step 2: Use *CDI* and *DAD* to calculate the non-carcinogenic risk (*HQ*_*i*_) respectively. The specific calculation formulas are given in Eqs. 10–12.


$$HQ_{i}=HQI_{i}+HQD_{i}$$ (10)

$$HQI_{i}=\frac{CDI}{RfD_{i}}$$ (11)

$$HQD_{i}=\frac{DAD}{RfD_{i}}$$ (12)

Where, *HQI*_*i*_ and *HQD*_*i*_ represent the non-carcinogenic risks of the i^th^ pollutant under ingestion and contact conditions, respectively. *RfD*_*i*_ is the reference value corresponding to the ingestion dose of the i^th^ pollutant.

Step 3: Sum the non-carcinogenic risks of all pollutants to obtain the total non-carcinogenic health risk index (HI). The calculation formula is given in Eq. 13.


$$HI=\sum_{i=1}^{n}HQ_{i}$$ (13)

## 4. Results and discussion

### 4.1. General hydrochemical characteristics

The hydrochemical parameters of groundwater sampled in the basin are listed in Table 1, along with the permissible limits set by World Health Organization (WHO) [61] and Chinese guidelines [62], as well as the standards for Natural Drinking Mineral Water [63] for comparison. The pH of groundwater ranges from 6.82 to 8.10, with an average of 7.78, and all samples meet the WHO guideline. The Total Hardness (TH) and Total Dissolved Solids (TDS) of groundwater range from 205.84 to 485.39 mg/L (mean 284.11 mg/L) and 282.00 to 1,263.83 mg/L (mean 456.72 mg/L), respectively. Only 3.85% of sampled groundwater exceeds the permissible limits of 450 mg/L for TH and 1000 mg/L for TDS set by the WHO guideline, indicating that most groundwater in the Tongde Basin is soft and fresh, suitable for drinking.

**Table 1:** Summary of groundwater physicochemical indexes within the Tongde basin.

Index	Unit	Min	Max	Mean	SD*	Guideline	% of the Sample Exceeding the Guideline
pH	/	6.82	8.10	7.78	0.29	6.5–8.5**	0.00%
TH	mg/L	205.84	485.39	284.11	56.74	450.00	3.85%
TDS	mg/L	282.00	1263.83	456.72	203.20	1000.00**	3.85%
K^+^	mg/L	0.56	18.20	4.33	3.73	/	/
Na^+^	mg/L	9.42	275.00	46.34	68.02	200.00	7.69%
Ca^2+^	mg/L	44.90	110.22	74.24	12.64	300.00**	38.46%
Mg^2+^	mg/L	9.72	69.26	23.99	12.88	100.00**	3.85%
Cl^-^	mg/L	4.76	187.89	39.33	46.57	250.00**	0.00%
SO_4_^2-^	mg/L	9.61	384.24	50.32	72.93	250.00**	3.85%
HCO_3_^-^	mg/L	246.00	537.00	326.11	76.23	/	/
NH_4_^+^	mg/L	0.00	1.42	0.16	0.33	0.20***	21.05%
NO_2_^-^	mg/L	0.00	0.08	0.01	0.02	0.02***	8.00%
NO_3_^-^	mg/L	1.72	42.86	9.80	9.37	50.00**	0.00%
Sr	mg/L	0.29	2.03	6.99	385.19	0.20****	100.00%
^87^Sr/^86^Sr	/	0.71	0.71	0.71	0.00	/	/

*Standard Deviation; ** WHO Guideline [61]; *** Chinese Guideline [62]; ****Drinking Natural Mineral Water [63].

Among major ions, Na^+^ and Ca^2+^ are the most abundant cations in the groundwater, with concentrations ranging from 9.42 to 275.00 mg/L (mean 74.24 mg/L) and 44.90 to 110.22 mg/L (mean 46.34 mg/L), respectively. The concentration of Mg^2+^ ranks second, ranging from 9.72 to 69.26 mg/L with a mean of 23.99 mg/L. K^+^ is the lowest major cation, with a range of 0.56 to 18.20 mg/L. Overall, except for K^+^, all major cations exceed the WHO guideline limits in 3.85% to 38.46% of the samples. The anions in groundwater are predominantly HCO_3_^-^, with contents ranging from 246.00 to 537.00 mg/L (mean 326.11 mg/L), followed by SO_4_^2-^ (9.61 to 384.24 mg/L, mean 50.32 mg/L) and Cl⁻ (4.76 to 187.89 mg/L, mean 39.33 mg/L). Only SO_4_^2-^ exceeds the WHO guideline of 250 mg/L in 3.85% of the samples.

Nitrogen concentrations were also determined for groundwater in the Tongde Basin to assess potential anthropogenic influences. As shown in Table 1, most groundwater samples exhibit very low nitrogen concentrations. However, NH_4_^+^ exceeds the Chinese guideline limits of 0.2 mg/L [62] in 21.05% of the samples, and NO_2_^-^ exceeds the Chinese guideline limit of 0.02 mg/L [62] in 8.00% of the samples. No groundwater has been observed with NO_3_^-^ concentration beyond the permissible limit of 50 mg/L for drinking recommended by WHO [61]. This indicates that only certain areas in the Tongde Basin are slightly affected by anthropogenic inputs, ensuring the overall suitability of groundwater for drinking purposes.

The special component contents of groundwater were determined. The concentration of Sr ranges from 0.29 to 2.03 mg/L, which is the most abundant and the only minor element meeting the minimum threshold (0.20 mg/L) of Drinking Natural Mineral Water [63], with a compliance rate of 100%. Additionally, to further analyze the source of Sr, the ^87^Sr/^86^Sr ratio was measured, with results averaging around 0.71.

### 4.2. Groundwater hydrochemical facies

**Fig 2:**
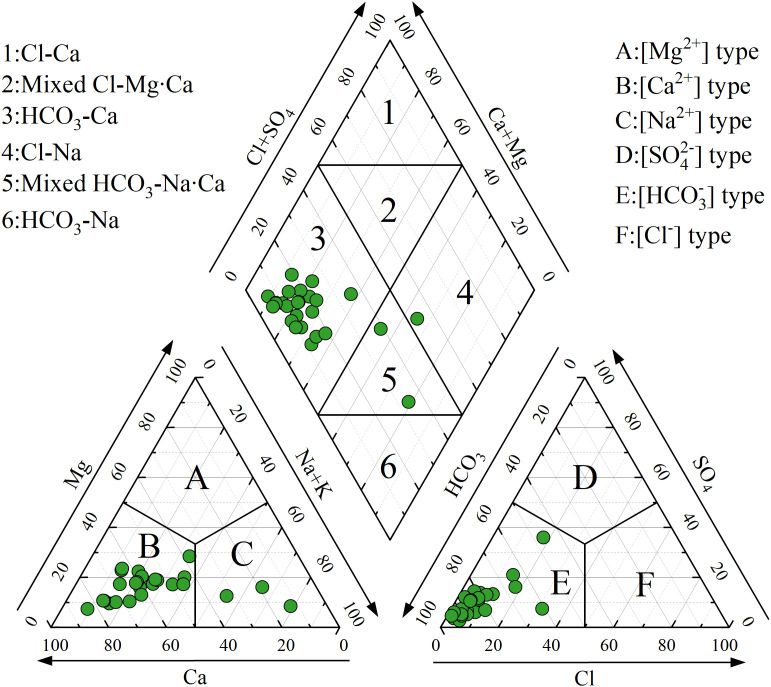
Piper trilinear diagram demonstrating groundwater hydrochemical composition within the study area.

Piper trilinear diagram is an effective tool for identifying groundwater hydrochemical facies and providing valuable insights into the potential evolution of groundwater hydrochemistry [64–66]. As shown in Fig 2, Ca^2+^ and HCO_3_^-^ are the dominant cations and anions, respectively. The majority of groundwater samples are classified as HCO_3_-Ca type. Only two samples are HCO_3_-Na·Ca type, and one sample is Cl-Na type, which may be associated with potential evaporation effects in some shallow locations. Overall, groundwater in the Tongde Basin exhibits a fresh hydrochemical facies of HCO_3_-Ca type, with no significant trend toward evolution into saltwater.

### 4.3. Mechanisms governing groundwater chemistry

#### 4.3.1. Major hydrochemistry.

Hydrochemical diagrams, correlation analysis, and the chlor-alkali index are used to identify the mechanisms governing the chemistry of strontium-rich groundwater in the Tongde Basin. Generally, the hydrochemical components of groundwater are primarily controlled by natural factors such as recharge water (usually precipitation), water-rock interactions, and evaporation [43,67–69]. Human activities further shape the hydrochemical composition of groundwater beyond these natural processes [70,71].

**Fig 3:**
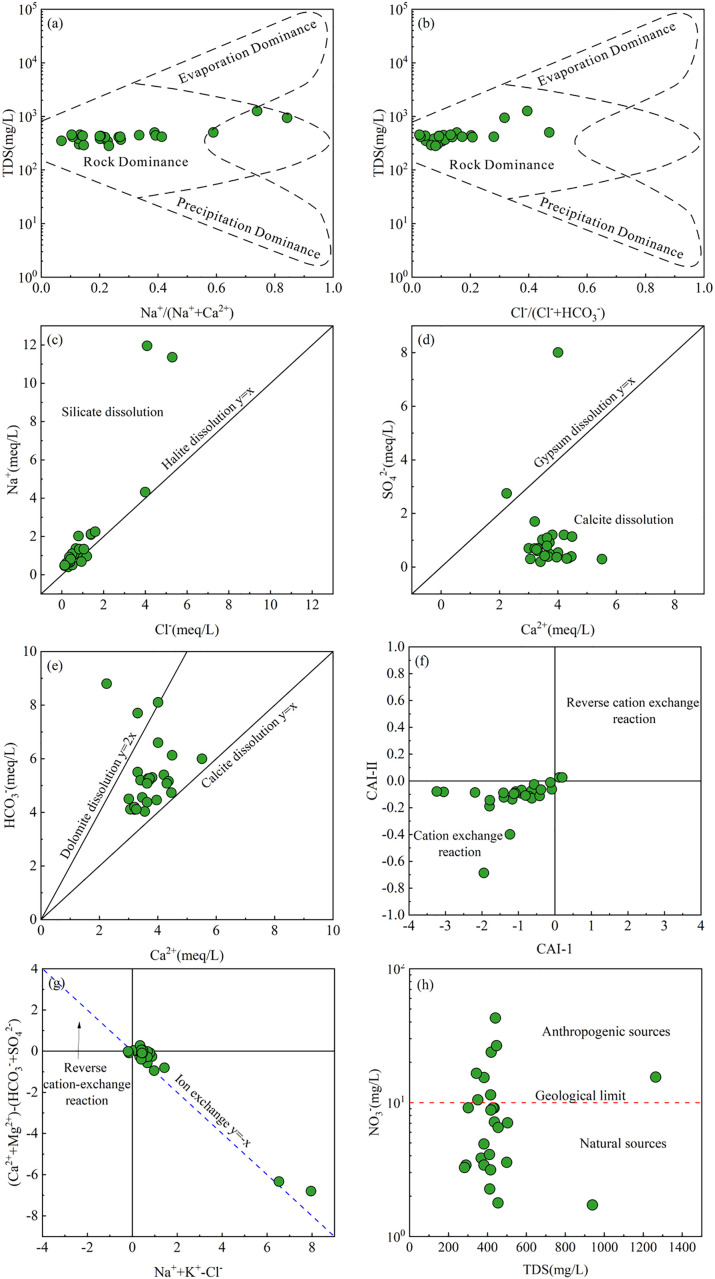
Scatter plots of (a) Na^+^/(Na^+^+Ca^2+^) versus TDS, and (b) Cl^-^/(Cl^-^ + HCO_3_^-^) versus TDS, (c) Na^+^ versus Cl^-^, (d) SO_4_^2-^ versus Ca^2+^, (e) HCO_3_^-^ versus Ca^2+^, (f) CAI-1 versus CAI-2, (g) (Na^+^+K^+^-Cl^-^) versus (Ca^2+^+Mg^2+^-HCO_3_^-^-SO_4_^2-^), and (h) NO_3_^-^ versus TDS of groundwater within the study area.

Gibbs diagrams, constructed using Na ⁺ /(Na⁺+Ca²⁺) or Cl^-^/(Cl^-^ + HCO₃^-^) versus TDS [72], are effective tools for visually identifying the major natural mechanisms governing the hydrochemical composition of natural waters [73,74]. As shown in Fig 3a, b, all sampled groundwaters fall within the rock-dominated zone of the Gibbs diagrams, indicating that water-rock interactions are the primary drivers of the hydrochemical composition of strontium-rich groundwaters in the Tongde Basin. Additionally, a few samples show a trend towards the evaporation-dominated zone, suggesting that evaporation also plays a role in shaping the hydrochemical characteristics to some extent.

**Fig 4:**
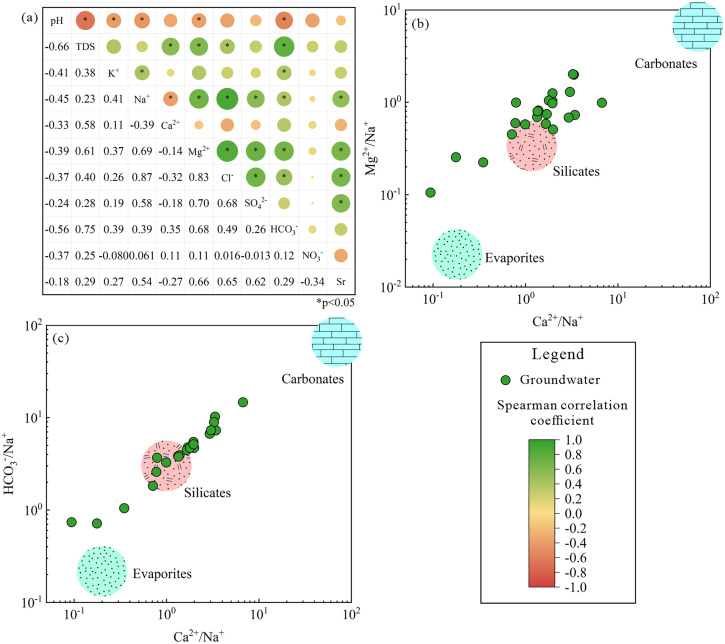
Groundwater hydrochemical analysis plots of (a) correlation coefficients between various physicochemical parameters, and end-member diagram of (b) Ca^2+^/Na^+^ versus Mg^2+^/Na^+^, and (c) Ca^2+^/Na^+^ versus HCO_3_^-^/Na^+^ within the study area.


The concentration ratios of various ions are used to further identify the principal sources of the hydrochemical constituents in groundwater. The ion ratio relationship between Na^+^ and Cl^-^ shows that most sampled groundwaters cluster close to the 1:1 ratio line, indicating that halite dissolution is the primary potential source of Na^+^ and Cl^-^ in the groundwaters (Fig 3c). However, some groundwaters significantly deviate from this line, showing anomalously high Na^+^ levels. This deviation suggests that these waters are influenced by cation exchange or silicate rock dissolution rather than halite dissolution. The weathering and dissolution of albite can increase both Na^+^ and HCO_3_^-^ concentrations in groundwater (Eq. 12). In Tongde basin, a positive correlation (correlation coefficient 0.39) between Na^+^ and HCO_3_^-^ in strontium-rich groundwater indicates potential silicate rock weathering (Fig 4a). Additionally, a negative correlation (correlation coefficient −0.39) between Na^+^ and Ca^2+^ suggests cation exchange may also affect Na^+^ concentrations in groundwater.

$$2NaAlSi_{3}O_{8}+2CO_{2}+11H_{2}O\to Al_{2}Si_{2}O_{5}(OH{)}_{4}+4H_{4}SiO_{4}+2Na^{+}+2HC{O_{3}}^{-}$$ (12)

In general, freshwater typically has low levels of SO_4_^2-^, which originates from the dissolution of gypsum and anhydrite. The ratio plot of SO_4_^2-^ versus Ca^2+^ shows that almost all sampled groundwaters in Tongde basin have higher Ca^2+^ milligram-equivalent concentrations compared to SO_4_^2-^ (Fig 3d). This indicates that gypsum is not the primary mineral determining the SO_4_^2-^ and Ca^2+^ contents in groundwater. Furthermore, a weak negative correlation exists between SO_4_^2-^ and Ca^2+^ (correlation coefficient −0.18, Fig 4a), suggesting these ions may come from other sources. In contrast, a stronger correlation between SO_4_^2-^ and Mg^2+^ (correlation coefficient 0.7, Fig 4a) implies that their concentrations in groundwater are likely controlled by similar hydrochemical processes. The study area has well-developed fracture structures, and pyrite and magnesite in these zones may be potential sources of SO_4_^2-^ and Mg^2+^ in groundwater.

All sampled groundwaters in Tongde basin fall between the calcite and dolomite dissolution lines on the scatter plot of HCO_3_^-^ versus Ca^2+^ (Fig 3e), indicating that the weathering and dissolution of calcite and dolomite contribute to the enrichment of Ca^2+^ and HCO_3_^-^ in groundwater. During dolomite dissolution, HCO_3_^-^ and equivalent amounts of Ca^2+^ and Mg^2+^ are released into the groundwater (Eq. 13). However, the groundwater shows a low negative correlation between Ca^2+^ and Mg^2+^ (correlation coefficient −0.14), inconsistent with dolomite dissolution (Fig 4a). Therefore, the Ca^2+^ in groundwater mainly originates from calcite, while the excess HCO_3_^-^ relative to Ca^2+^ results from silicate weathering and dissolution rather than dolomite. End-member diagrams based on Mg^2+^/Na^+^ and HCO_3_^-^/Na^+^ versus Ca^2+^/Na^+^ were constructed to identify the major rocks (including evaporites, silicates, and carbonates) involved in water-rock interactions in the aquifer [75]. As shown in Fig 4b, c, most sampled groundwaters plot in the silicate-dominated zone, with only a few in the evaporite-dominated zone. This confirms that silicate dissolution is the primary process governing the hydrochemical composition of strontium-rich groundwaters in the Tongde Basin, while evaporite dissolution also plays a significant role in groundwater hydrochemistry in some locations.


$$CaMg{\left(CO_{3}\right)}_{2}+2CO_{2}+2H_{2}O\to Ca^{2+}+Mg^{2+}+4HC{O_{3}}^{-}$$ (13)

As mentioned above, cation exchange is a potential process controlling the hydrogeochemical evolution of groundwater. The chloro-alkaline indices (CAI-I and CAI-II) (Eqs. 14 and 15) based on the ratio of milligram equivalent concentrations of ions are introduced to further substantiate this potential process in the aquifer. When both CAI-I and CAI-II results are negative, it indicates the occurrence of positive cation exchange interactions in the groundwater (Eq. 16). Conversely, if there is a reverse cation exchange process, both CAI-I and CAI-II results will be positive (Eq. 17). Nearly 92% of the sampled groundwaters in the study area exhibit negative CAI results (Fig 3f), indicating that a positive cation exchange process occurs in the aquifer. Furthermore, the excess of Na^+^ and the deficiency of Ca^2+^ and Mg^2+^ in the groundwater also signify that a positive cation exchange process has occurred in the aquifer, leading to the replacement of Ca^2+^ and Mg^2+^ by Na^+^ (Fig 3g).


$$CAI-I=\frac{Cl^{-}-(Na^{+}+K^{+})}{Cl^{-}}$$ (14)

$$CAI-II=\frac{Cl^{-}-(Na^{+}+K^{+})}{HC{O_{3}}^{-}+S{O_{4}}^{2-}+C{O_{3}}^{2-}+N{O_{3}}^{-}}$$ (15)

$$Ca^{2+}(orMg^{2+})+2NaX(solids)\to 2Na^{+}+CaX_{2}(orMgX_{2})(solids)$$ (16)

$$2Na^{+}+CaX_{2}(orMgX_{2})(solids)\to Ca^{2+}(orMg^{2+})+2NaX(solids)$$ (17)

The shallow depths of the phreatic groundwater in the study area and the open groundwater system make the hydrochemical components of groundwater susceptible to a range of anthropogenic activities in addition to natural processes. NO_3_^-^ contamination from various sources is prevalent in groundwater environments worldwide, and its concentration has become a crucial indicator for assessing whether human activities have impacted the hydrochemical quality of groundwater [76]. When NO_3_^-^ concentrations are below 10 mg/L, their levels are primarily controlled by geological factors [71]. Conversely, concentrations exceeding 10 mg/L indicate the presence of anthropogenic inputs of NO_3_^-^ pollution [77]. In the study area, 32% of the sampled groundwater exhibited NO_3_^-^ concentrations exceeding 10 mg/L (Fig 3h), indicating the involvement of anthropogenic activities in shaping the hydrochemical components of groundwater in the study area. The extensive agriculture and animal husbandry within the basin may be the major anthropogenic sources of nitrate contaminants in the groundwater environment.

Overall, the hydrochemical composition of strontium-rich groundwater in the Tongde Basin is controlled by both natural processes and anthropogenic factors. The ion composition of groundwater is primarily dominated by the weathering of silicates, such as feldspar in the sedimentary sandstones within the basin, and cation exchange processes. Additionally, the dissolution of magnesite, pyrite, and calcite also contributes to groundwater hydrochemical composition. The development of agriculture and animal husbandry are the main reasons for the enrichment of nitrates in some groundwaters.

#### 4.3.2. Beneficial components.

**Fig 5:**
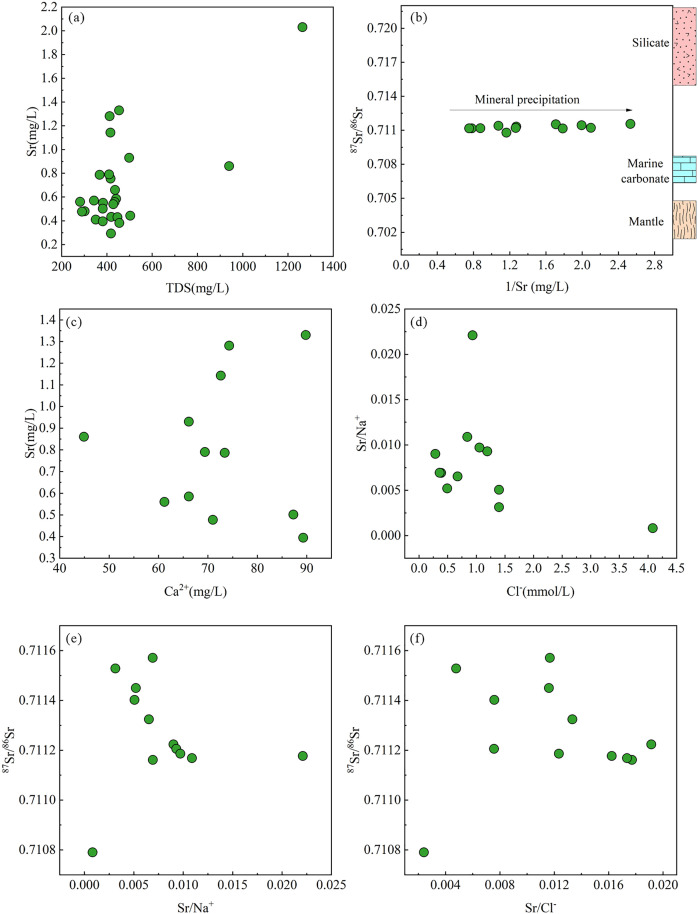
Scatter plots of (a) Sr content versus TDS, (b) 87Sr/86Sr versus 1/Sr, (c) Sr versus Ca^2+^, (d) Sr/Na^+^ versus Cl^-^, (e) ^87^Sr/^86^Sr versus Sr/Na^+^, (f) ^87^Sr/^86^Sr versus Sr/Cl^-^ of groundwater within the Tongde Basin.

In the Tongde Basin, the strontium (Sr) concentration in groundwater ranges from 0.29 to 2.03 mg/L, with relatively high overall content, making it a dominant component in groundwater. Groundwater samples with higher Sr concentrations also exhibit relatively higher total dissolved solids (TDS), showing a certain linear positive correlation (Fig 5a). This suggests that the increase in Sr concentration in groundwater may be related to evaporation or water–rock interactions processes [78].

The relationship between Sr isotopes (^87^Sr/^86^Sr) and other physicochemical characteristics of groundwater is an effective means to explore the formation mechanism of dominant components in groundwater. The relationship between ^87^Sr/^86^Sr and 1/Sr reveals the source of dominant components in groundwater of the Tongde Basin. The values of ^87^Sr/^86^Sr in the basin range from 0.7108 to 0.7116. These values are higher than those in carbonate leaching solutions (0.709) [79] but lower than those in silicate leaching solutions (0.715) [80]. All samples are distributed between the silicate and marine carbonate end-members (Fig 5b), indicating that the groundwater within the Tongde Basin is affected by a mixing process from the dissolution of silicates and carbonates. Therefore, Sr, as the beneficial component in the basin groundwater, likely originates from water–rock interactions with silicate and carbonate rocks, rather than from evaporative processes.

Strontium (Sr) and calcium (Ca^2+^) share similar hydrogeochemical properties and are commonly found in abundance in carbonate and silicate rocks. The relationship between Sr and Ca^2+^ can indicate the source of Sr. An strong positive correlation between Sr and Ca^2+^ suggests that Sr originates from the dissolution of carbonate minerals [81]. As shown in Fig 5c, there is a weak positive correlation between Sr and Ca^2+^ for groundwater within the basin. This indicates that while the dissolution of carbonate minerals does provide a portion of the Sr in groundwater, the remaining Sr is derived from other sources. Given that the primary source of Ca²⁺ in groundwater within the basin is the dissolution of calcite, the Sr from carbonate rocks is likely derived from the dissolution of strontianite and the substitution of Sr for Ca^2+^ in calcite [82].

The other source of Sr in groundwater of the Tongde Basin is revealed through the relationship between Sr/ Na^+^ and Cl^-^. As shown in Fig 5d, the groundwater within the basin exhibits a negative correlation between Sr/ Na^+^ and Cl^-^, indicating that another source of Sr is sodium silicate minerals, primarily dominated by albite [83]. These Sr atoms enter the mineral lattice by substituting for Na^+^ positions within the mineral structure and are subsequently released into groundwater through water–rock interactions. Similarly, if Sr in groundwater had a single source, the ratios of ⁸⁷Sr/⁸⁶Sr to Sr/ Na^+^ and Sr/ Cl^-^ should show a clear linear positive or negative correlation [84]. However, no significant linear relationship is observed in Fig 5e and 5f, indicating the diversity of Sr sources in the groundwater. The rock formations exposed in the peripheral mountainous areas of the basin are mainly arkosic sandstone and argillaceous siltstone. These rocks contain significant amounts of feldspar, quartz, and other silicate minerals, as well as carbonate minerals present as cementing materials [85]. Overall, the Sr in the Tongde Basin originates from the dissolution of silicate minerals and carbonate cements in arkosic sandstone and argillaceous siltstone, with the minerals involved in dissolution including albite, calcite, and strontianite.

### 4.4. Groundwater exploitation suitability for drinking purpose

#### 4.4.1. Overall groundwater quality based on EWQI assessment.

**Fig 6:**
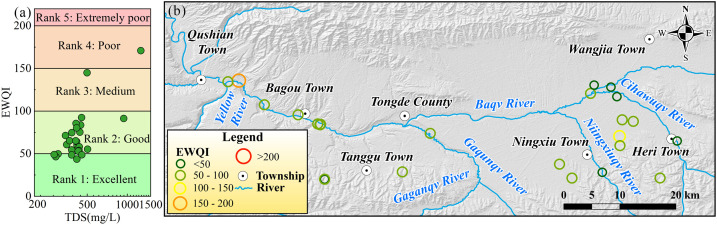
Visualization of groundwater quality, including (a) scatter plots of EWQI value versus TDS, (b) spatial distribution of groundwater quality based on EWQI assessment within the Tongde Basin. (The map was created via ArcGIS 10.2 (https://www.esri.com/en-us/arcgis/products/arcgis-desktop/resources) based on the publicly available ASTER GDEMV2 data from Geospatial Data Cloud site, Computer Network Information Center, Chinese Academy of Sciences (http://www.gscloud.cn)).

Parameters of Na^+^, K^+^, Ca^2+^, Mg^2+^, HCO_3_^-^, SO_4_^2-^, Cl^-^, TDS, TH, Sr, NH_4_^-^, NO_2_^-^ and NO_3_^-^ are selected as the basis variables for estimating the overall water quality of groundwater within the Tongde Basin based on the Entropy-weighted Water Quality Index (EWQI). The EWQI value of G24 is the highest at 170.83, while G9 has the lowest value at 43.58 (Fig 6a). The average EWQI value of sampled groundwaters within the basin is 69.12. Among them, 23% of the sampled groundwaters have an EWQI value below 50, falling into the excellent category (Fig 6a). The majority (69%) of the samples are in the good category. There is one sample each in the medium and poor categories, with no samples in the extremely poor category. Overall, the groundwater quality in the Tongde Basin is relatively good, with most samples in the good category. The positive correlation between EWQI and TDS (Fig 6a) further indicates that changes in groundwater quality are closely related to factors causing an increase in TDS.

As shown in Fig 6b, the distribution of EWQI for groundwater in the basin is relatively uniform, generally ranging between 50 and 100. Groundwater samples classified as excellent are primarily found in the upstream area. Agricultural activities in this region can alter the hydrochemical composition of groundwater (e.g., G6), leading to higher EWQI values. The groundwater sample with the highest EWQI (G24) is located in the downstream area. This sample’s hydrochemical characteristics are marked by significantly higher concentrations of certain ions (Mg^2+^, Na^+^, K^+^, HCO_3_^-^, SO_4_^2-^ and Cl^-^) compared to other samples, likely due to prolonged water-rock interactions.

#### 4.4.2. Potential health risk of dissolved harmful substances.

**Fig 7:**
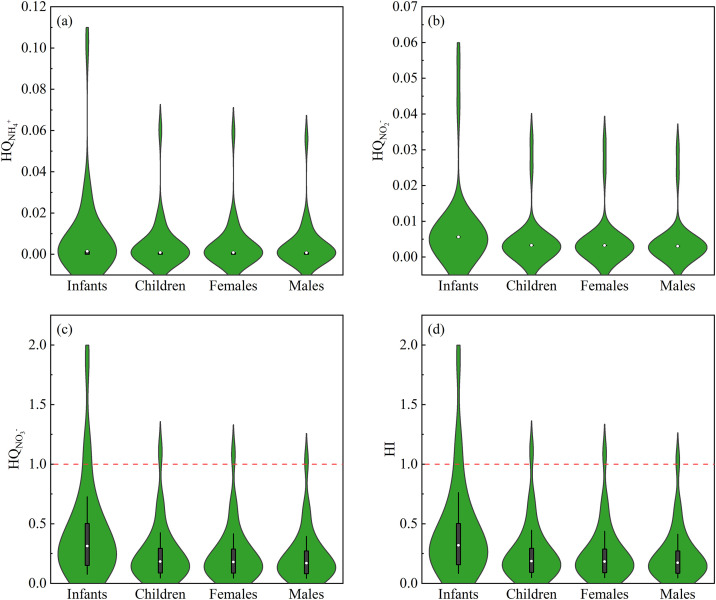
The violin plots of HQ posed by (a) NH_4_^+^, (b) NO_2_^-^, (c) NO_3_^-^, and (d) HI of groundwater within the Tongde Basin.

The non-carcinogenic health risk assessment results for the four population groups in the study area show that infants are at the highest risk, with HI values ranging from 0.08 to 1.89 and an average of 0.45 (Fig 7). Following infants are children, adult females, and adult males, with average HI values of 0.26, 0.26, and 0.24, respectively. In comparison, the average HI values for children, adult females, and adult males are relatively close, with only the HI value for infants being significantly higher. This is attributed to the immature immune system of infants, which is less tolerant to toxic substances [42,53]. Additionally, the maximum HI values for all four population groups exceed 1, indicating that some individuals in the study area are facing non-negligible non-carcinogenic risks [86].

As shown in Fig 7, NO_3_^-^ poses the highest non-carcinogenic risk to all four population groups, with average HQ values of 0.43, 0.25, 0.25, and 0.23 for infants, children, adult females, and adult males, respectively. In contrast, the average HQ values for NH_4_^+^ and NO_2_^-^ do not exceed 0.01 for any group, indicating that these components have a very limited impact on non-carcinogenic risks for local residents. The results are clearly visualized in Fig 7, showing that some HQ values for NO₃ ⁻ exceed the acceptable limit of 1. In summary, NO₃ ⁻ is the primary cause of increased non-carcinogenic risks for residents within the basin.

**Fig 8:**
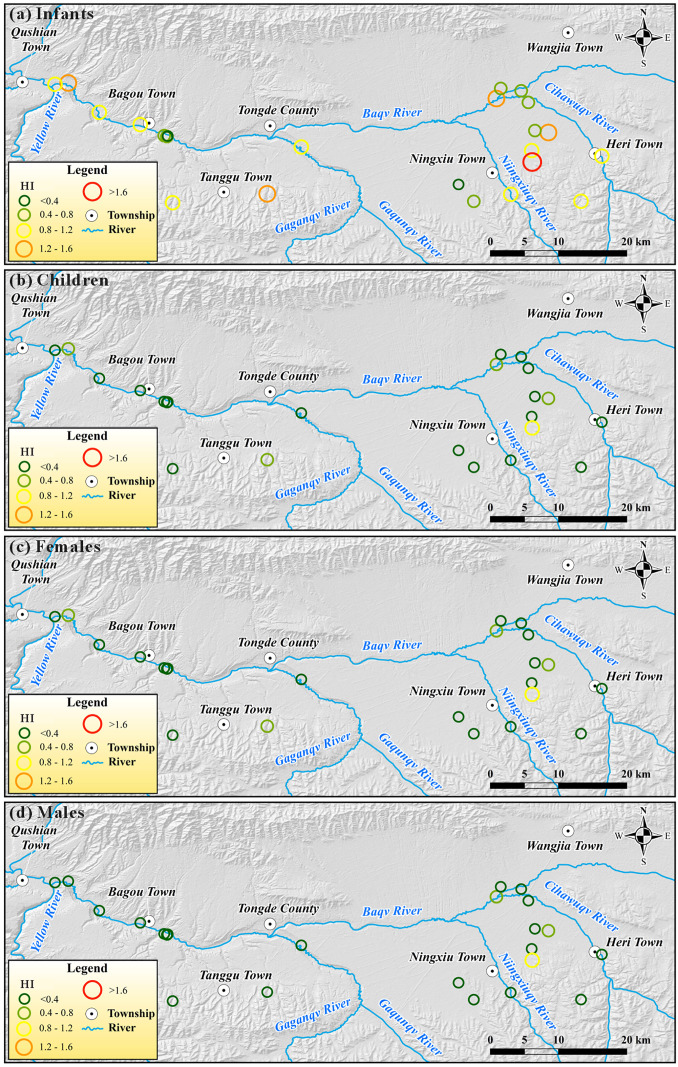
Spatial distribution of overall non-carcinogenic risk (HI) for (a) infants, (b) Children, (c) adult females, and (d) adult males posed by potential harmful substances of groundwater within the Tongde Basin. (The map was created via ArcGIS 10.2 (https://www.esri.com/en-us/arcgis/products/arcgis-desktop/resources) based on the publicly available ASTER GDEMV2 data from Geospatial Data Cloud site, Computer Network Information Center, Chinese Academy of Sciences (http://www.gscloud.cn)).

It can be seen from Fig 8, groundwater with high HI values is mainly distributed in the upstream area of the basin. This is primarily due to large-scale farms in the upstream areas, where the use of fertilizers and pesticides increases the content of specific harmful substances in groundwater, thereby posing a risk to human health. Compared to the high HI values for infants, the distribution of HI values for children, adult females, and adult males is more uniform and generally falls within the acceptable range. Intense human activities have not caused significant non-carcinogenic risks for these three groups. Furthermore, the HI for infants in some downstream areas of the basin remains high. This is due to the presence of a few scattered farmlands around these groundwater samples. Although the agricultural activities are relatively minor compared to those in the upstream area, their impact on groundwater is still significant and cannot be ignored.

## 5. Conclusions

The present research focuses on the natural mineral groundwater in alpine regions. The Tongde basin on Tibetan Plateau has been taken as an example to explore the hydrochemical characteristics and formation mechanisms of natural mineral groundwater in alpine basins and its suitability for sustainable development and exploitation under human activities. The main findings are as below:

(1) Groundwater in the alpine Tongde Basin on Tibetan Plateau belongs to strontium-rich mineral water, with Sr concentration ranging from 0.29 to 2.03 mg/L, exceeding the mineral water threshold of 0.20 mg/L. Groundwater across the basin is neutral to slightly alkaline, with pH values ranging from 6.82 to 7.78 and an average of 7.78. It has a relatively large variation in salinity with TDS varying from 282.00 mg/L to 1,263.83 mg/L, but predominantly fresh with the average TDS of 456.72 mg/L and approximately 96.15% of sampled groundwaters having the TDS below 1,000 mg/L. Groundwater is featured by fresh hydrochemical facies of HCO_3_-Ca type throughout the basin, but also by saltier types of HCO_3_-Na·Ca and Cl-Na at some sporadic sites. NH_4_^+^ and NO_2_^-^ exceed the Chinese drinking water permissible limits of 0.2 mg/L and 0.02 mg/L, respectively, in groundwater at 21.05% and 8% of sampling sites. Although the NO_3_^-^ concentration is within the WHO-recommended drinking water limit of 50 mg/L at all sampling sites, it exceeds the natural NO_3_^-^ limit of 10 mg/L at many sites, with a maximum concentration of 42.86 mg/L.
(2) Groundwater in Tongde Basin is primarily governed by natural mechanisms of water-rock interactions. Evaporation also plays a role in shaping the groundwater hydrochemical composition at some sporadic sites, though its influence is limited. Groundwater hydrochemical components are predominantly originated from silicates weathering and cation exchange process, along with the dissolution of magnesite, pyrite, and calcite. The enriched Sr in groundwater derived from the dissolution of silicate minerals and carbonate cements in arkosic sandstone and argillaceous siltstone, with albite, calcite, and strontianite being the primary minerals involved. Agricultural practices and animal husbandry within the basin have significantly altered groundwater chemistry, introducing nitrogen contaminants in the form of NH_4_^+^, NO_2_^-^ and NO_3_^-^.
(3) Groundwater in the basin has a relatively large variation in hydrochemical quality with the EWQI value ranging from 43.58 to 170.83, but is in excellent to good quality in most sites. Only two sampled groundwaters are with the quality varying from medium (site G6) to poor (site G24) categories. Agricultural practices and the prolonged water-rock interaction are primarily responsible for these two medium to poor quality groundwaters. The nitrogen contaminants would pose potential non-carcinogenic health risk with the total non-carcinogenic health risk index (HI) beyond the permissible limit of 1 to all populations. Although levels of NH_4_^+^ and NO_2_^-^ in groundwater exceed Chinese drinking water permissible limits, they generally do not pose significant health hazards to humans. However, the elevated concentrations of NO_3_^-^ can pose health risks, particularly to the infants. Agricultural contaminants are a significant source of potential health risks for groundwater and must be addressed in the development of alpine basins, especially those with the endowment of mineral water resources. Precision nutrient management, grounded in optimized fertilization strategies, is essential to mitigate nitrogen leaching from agricultural non-point sources into aquifers. Concurrently, proactive surveillance of geogenic trace elements is critical for evidence-based, risk-oriented protection of drinking water quality.

## Supporting information

S1 Table The exposure parameters and RfD_oral_ used in human health risk assessment. The exposure factors include Exposure Frequency (EF, d/y), Exposure Duration (ED, years), Ingestion Rate (IR, L/d), Body Weight (BW, kg), Skin Surface Area (SA, cm^2^), Averaging Time (AT, h), Exposure Time (T, h), Event Frequency (EV, day), Volumetric Conversion Factor (CF, L/cm^3^), and Dermal Permeability Coefficient (K_p_, cm/h). Each exposure factor lists the values for four demographic groups (Infants, Children, Adult Females, and Adult Males). The corresponding oral reference doses (RfD_oral_, mg/(kg × day)) for the contaminants of concern (NO_3_^-^, NO_2_^-^, NH_4_^+^) are listed alongside.
(DOCX)

## References

[1] Elena F, Vasiliy L, Natalia K, Arslan S, Elena M, Ekaterina B, et al. Hydrogeology and hydrogeochemistry of mineral sparkling groundwater within Essentuki area (Caucasian mineral water region). Environ Earth Sci. 2019;79(1). doi: 10.1007/s12665-019-8721-2

[2] Hu F, et al. Characteristics of hydrochemical distribution and source analysis of threshold value elements of mineral water in Huangshui River catchment. Bulletin of Geological Science and Technology. 2025;44(3):309–23.

[3] Kong F, Xue Y, Qiu D, Su M, Gong H, Wang X. Assessment of the Mineralization Processes of Potable Natural Mineral Water Using the Chemical Thermodynamics Analysis. Pol J Environ Stud. 2021;31(1):121–34. doi: 10.15244/pjoes/139737

[4] Wang L, Xiao Y, Yang H, Zhang Y, Wang S, Qi Z, et al. Formation mechanism of high-altitude glacial mineral water in the Kunlun Mountains of Tibetan Plateau: Insights from isotopes and hydrochemistry. Journal of Hydrology: Regional Studies. 2024;53:101789. doi: 10.1016/j.ejrh.2024.101789

[5] Yu Y, Bian J, Ma Y, Li Y, Li J. Formation mechanism of hydrogeochemical characterization of mineral water in Antu County, Changbai Mountain area. Environ Sci Pollut Res Int. 2022;29(49):73910–25. doi: 10.1007/s11356-022-20380-1 35624376

[6] Arab S, Alshikh A. Estimating of some trace elements in mineral water in the Kingdom of Saudi Arabia. Life Science Journal-Acta Zhengzhou University Overseas Edition. 2010;7(3):133–7.

[7] Grebenshchikova V, Kuzmin M, Novopashina A, Kuz’mina E. Distribution and role of fluorine in the aquatic ecosystem (mineral springs, groundwater, tributaries, Baikal water, and the Angara water source) of lake Baikal, Russia. China Geology. 2025;8(0):1–11. doi: 10.31035/cg20230100

[8] Petraccia L, Liberati G, Masciullo SG, Grassi M, Fraioli A. Water, mineral waters and health. Clin Nutr. 2006;25(3):377–85. doi: 10.1016/j.clnu.2005.10.002 16314004

[9] Yan B, Xiao C, Liang X, Wu S. Hydrogeochemical tracing of mineral water in Jingyu County, Northeast China. Environ Geochem Health. 2016;38(1):291–307. doi: 10.1007/s10653-015-9719-7 26040975

[10] Ma B-h., Liu S, Wang X-z., Zhai X, Li H-c. A preliminary study on the spatial distribution characteristics and causes of strontium-rich mineral water in the Dushan complex. Journal of Groundwater Science and Engineering. 2018;6(21):48–58. doi: 10.19637/j.cnki.2305-7068.2018.02.005

[11] Qi Z, et al. Origin of mineral water in Heicigou, northern slope of eastern Kunlun Mountains. Journal of Salt Lake Research. 2024;32(3):21–31.

[12] Zhu X, et al. Distribution and characterization analyses of strontium-bearing mineral spring water in the Chengde region. Hydrogeology & Engineering Geology. 2020;47(6):65–73. doi: 10.16030/j.cnki.issn.1000-3665.202009007

[13] Bian J, Li Y, Ma Y, Li J, Yu Y, Sun W. Study on Hydrochemical Characteristics and Formation Process of Antu Mineral Water in Changbai Mountain, China. Water. 2022;14(18):2770. doi: 10.3390/w14182770

[14] Wang G, Xiao C, Qi Z, Lai Q, Meng F, Liang X. Research on the exploitation and utilization degree of mineral water based on ecological base flow in the Changbai Mountain basalt area, northeast China. Environ Geochem Health. 2022;44(7):1995–2007. doi: 10.1007/s10653-021-00865-7 33661451

[15] Balderer WP, Piatti E. The St. Moritz Mauritius mineral spring (Upper Engadine Valley, SE Switzerland): review of its importance by the joint facts of geological occurrence, archeology, health effects, chemical properties, and long-term chemical stability. Environ Earth Sci. 2023;82(9). doi: 10.1007/s12665-023-10800-x

[16] Baranovskaya EI, Kharitonova NA, Chelnokov GA, Tarasenko IA, Maslov AA. Chemical and Isotopic Features of a High pCO2 Natural Mineral Water from Essentuki Field (Caucasian Mineral Water Region, Russia). Water. 2023;15(5):901. doi: 10.3390/w15050901

[17] Hegler F, Lösekann-Behrens T, Hanselmann K, Behrens S, Kappler A. Influence of seasonal and geochemical changes on the geomicrobiology of an iron carbonate mineral water spring. Appl Environ Microbiol. 2012;78(20):7185–96. doi: 10.1128/AEM.01440-12 22865064 PMC3457109

[18] Kis B-M, Baciu C, Zsigmond A-R, Kékedy-Nagy L, Kármán K, Palcsu L, et al. Constraints on the hydrogeochemistry and origin of the CO2-rich mineral waters from the Eastern Carpathians – Transylvanian Basin boundary (Romania). Journal of Hydrology. 2020;591:125311. doi: 10.1016/j.jhydrol.2020.125311

[19] Rashid A, Farooqi A, Gao X, Zahir S, Noor S, Khattak JA. Geochemical modeling, source apportionment, health risk exposure and control of higher fluoride in groundwater of sub-district Dargai, Pakistan. Chemosphere. 2020;243:125409. doi: 10.1016/j.chemosphere.2019.125409 31778919

[20] Vinograd N, Porowski A. Application of isotopic and geochemical studies to explain the origin and formation of mineral waters of Staraya Russa Spa, NW Russia. Environ Earth Sci. 2020;79(8). doi: 10.1007/s12665-020-08923-6

[21] Zhang H, Yang Y, Qi H, Lu Y, Yu T. Hydrochemical evolution of rare cold mineral waters in the Wudalianchi UNESCO Global Geopark, China. Environ Earth Sci. 2018;77(10). doi: 10.1007/s12665-018-7543-y

[22] Hyun-Tae K, Kim TS. Analysis of brand naming and marketing implications of mineral water sold in China: Jeju SamDaSoo, Evian, and Tibet Spring 5100. Humanities Review. 2015;36:81–98.

[23] Lv G, Zhang Y, Liu J, Yang M, Wang S. Geochemical characteristics, Li source and genesis mechanism of thermal mineral water in Sichuan Basin, SW China. Geothermics. 2024;122:103079. doi: 10.1016/j.geothermics.2024.103079

[24] Li Y, Bian J, Li J, Ma Y, Anguiano JHH. Hydrochemistry and stable isotope indication of natural mineral water in Changbai Mountain, China. Journal of Hydrology: Regional Studies. 2022;40:101047. doi: 10.1016/j.ejrh.2022.101047

[25] XIA H, YANG L, FENG Q, SU Y, ZOU X, HE W. Progress of Research on the Impact of Climate Change on Hydrological Processes in Cold Regions in Alpine Mountains of Western China. JSLR. 2025;33(4):12–25. doi: 10.3724/j.yhyj.2025026

[26] Xiao Y, Liu K, Zhang Y, Yang H, Wang S, Qi Z, et al. Numerical investigation of groundwater flow systems and their evolution due to climate change in the arid Golmud river watershed on the Tibetan Plateau. Front Earth Sci. 2022;10. doi: 10.3389/feart.2022.943075

[27] Wang S, Wang F, Xiao Y, Dai S. Distribution and Utilization of Natural Mineral Water Resources in Qinghai Province of China. Beijing: Geological Publishing House; 2024.

[28] Luo Y, Xiao Y, Hao Q, Zhang Y, Zhao Z, Wang S, et al. Groundwater geochemical signatures and implication for sustainable development in a typical endorheic watershed on Tibetan plateau. Environ Sci Pollut Res Int. 2021;28(35):48312–29. doi: 10.1007/s11356-021-14018-x 33905060

[29] Wang W, Zhang S, Wang S, Zhang C, Zhang G, Wang J, et al. Spatial Variations and Regulating Processes of Groundwater Geochemistry in an Urbanized Valley Basin on Tibetan Plateau. Applied Sciences. 2024;14(21):9804. doi: 10.3390/app14219804

[30] Xiao Y, Hu W, Zhang Y, Zhao Z, Qin G, Chen H, et al. Spatiotemporal variation and driving forces of soil salinization in the lower reach of arid endorheic basins: Critical role of lake system and groundwater overflow. Agricultural Water Management. 2025;315:109568. doi: 10.1016/j.agwat.2025.109568

[31] Xiao Y, Shao J, Frape SK, Cui Y, Dang X, Wang S, et al. Groundwater origin, flow regime and geochemical evolution in arid endorheic watersheds: a case study from the Qaidam Basin, northwestern China. Hydrol Earth Syst Sci. 2018;22(8):4381–400. doi: 10.5194/hess-22-4381-2018

[32] Yang H, Xiao Y, Yang S, Zhao Z, Wang S, Xiao S, et al. Geochemical fingerprints, evolution, and driving forces of groundwater in an alpine basin on Tibetan Plateau: Insights from unsupervised machine learning and objective weight allocation approaches. Journal of Hydrology: Regional Studies. 2024;56:102054. doi: 10.1016/j.ejrh.2024.102054

[33] Yang S, Zhao Z, Wang S, Xiao S, Xiao Y, Wang J, et al. Hydrogeochemical Insights into the Sustainable Prospects of Groundwater Resources in an Alpine Irrigation Area on Tibetan Plateau. Sustainability. 2024;16(21):9229. doi: 10.3390/su16219229

[34] Bityukova L, Petersell V. Chemical composition of bottled mineral waters in Estonia. Journal of Geochemical Exploration. 2010;107(3):238–44. doi: 10.1016/j.gexplo.2010.07.006

[35] Mwiathi NF, Gao X, Li C, Rashid A. The occurrence of geogenic fluoride in shallow aquifers of Kenya Rift Valley and its implications in groundwater management. Ecotoxicology and Environmental Safety. 2022;229:113046. doi: 10.1016/j.ecoenv.2021.113046 34875514

[36] Ullah Z, Younas F, Bacha AUR, Rashid A, Al-Onazi WA, Sardar MF. Occurrence of toxic elements in river areas along drains and groundwater resources: source of contamination and associated health risk. Environ Monit Assess. 2024;196(5):480. doi: 10.1007/s10661-024-12648-5 38676764

[37] Yan B, Liang X, Xiao C. Hydrogeochemical Characteristics and Genesis Model of Jinjiang and Julong Hot Springs in Changbai Mountain, Northeast China. Geofluids. 2018;2018:1–16. doi: 10.1155/2018/1694567

[38] Hao Q, Li Y, Xiao Y, Yang H, Zhang Y, Wang L, et al. Hydrogeochemical fingerprint, driving forces and spatial availability of groundwater in a coastal plain, Southeast China. Urban Climate. 2023;51:101611. doi: 10.1016/j.uclim.2023.101611

[39] Rashid A, Ayub M, Bundschuh J, Gao X, Ullah Z, Ali L, et al. Geochemical control, water quality indexing, source distribution, and potential health risk of fluoride and arsenic in groundwater: Occurrence, sources apportionment, and positive matrix factorization model. J Hazard Mater. 2023;460:132443. doi: 10.1016/j.jhazmat.2023.132443 37666175

[40] Rashid A, Ayub M, Javed A, Khan S, Gao X, Li C, et al. Potentially harmful metals, and health risk evaluation in groundwater of Mardan, Pakistan: Application of geostatistical approach and geographic information system. Geoscience Frontiers. 2021;12(3):101128. doi: 10.1016/j.gsf.2020.12.009

[41] Rashid A, Guan D-X, Farooqi A, Khan S, Zahir S, Jehan S, et al. Fluoride prevalence in groundwater around a fluorite mining area in the flood plain of the River Swat, Pakistan. Sci Total Environ. 2018;635:203–15. doi: 10.1016/j.scitotenv.2018.04.064 29660723

[42] Saraswat A, Ram S, Raza MB, Islam S, Sharma S, Omeka ME, et al. Potentially toxic metals contamination, health risk, and source apportionment in the agricultural soils around industrial areas, Firozabad, Uttar Pradesh, India: a multivariate statistical approach. Environ Monit Assess. 2023;195(7):863. doi: 10.1007/s10661-023-11476-3 37336819

[43] Ullah Z, Talib MA, Rashid A, Ghani J, Shahab A, Irfan M, et al. Hydrogeochemical Investigation of Elevated Arsenic Based on Entropy Modeling, in the Aquifers of District Sanghar, Sindh, Pakistan. Water. 2021;13(23):3477. doi: 10.3390/w13233477

[44] Ullah Z, Zeng X-C, Rashid A, Ghani J, Ali A, Shah M, et al. Integrated approach to hydrogeochemical appraisal of groundwater quality concerning arsenic contamination and its suitability analysis for drinking purposes using water quality index. Sci Rep. 2023;13(1):20455. doi: 10.1038/s41598-023-40105-9 37993472 PMC10665467

[45] Rashid A, Ayub M, Gao X, Khattak SA, Ali L, Li C, et al. Hydrogeochemical characteristics, stable isotopes, positive matrix factorization, source apportionment, and health risk of high fluoride groundwater in semiarid region. J Hazard Mater. 2024;469:134023. doi: 10.1016/j.jhazmat.2024.134023 38492393

[46] Xiao Y, Zhang Y, Yang H, Wang L, Han J, Hao Q, et al. Interaction regimes of surface water and groundwater in a hyper-arid endorheic watershed on Tibetan Plateau: Insights from multi-proxy data. Journal of Hydrology. 2024;644:132020. doi: 10.1016/j.jhydrol.2024.132020

[47] Yu L, Feng G, Liu Q, Tang C, Wu B, Mao P, et al. Assessment of natural radioactivity and consequent radiological hazard in different brands of commercialized bottled mineral water produced in China. J Water Health. 2020;18(4):566–73. doi: 10.2166/wh.2020.038 32833682

[48] Hao Q, Lu C, Zhu Y, Xiao Y, Gu X. Numerical investigation into the evolution of groundwater flow and solute transport in the Eastern Qaidam Basin since the Last Glacial Period. Geofluids. 2018;2018.

[49] Zhang Y, Li Q, Luo Y, Yan L, Peng K, Liu Z, et al. Groundwater salinization in a subtropical region, Beihai, southern China: Insights from hydrochemistry and multiple isotopes (H, O, S, Sr). Applied Geochemistry. 2022;141:105323. doi: 10.1016/j.apgeochem.2022.105323

[50] Liu K, Yan H, Wang W, Wang S, Wang Z, Xiao Y, et al. Hydrogeochemical signatures, quality and driving forces of phreatic groundwater in a typical headwater region of the Yellow River watershed. Appl Water Sci. 2025;15(5). doi: 10.1007/s13201-025-02458-6

[51] Hao Q, Xiao Y, Liu K, Yang H, Chen H, Wang L, et al. Spatial pattern of groundwater chemistry in a typical piedmont plain of Northern China driven by natural and anthropogenic forces. Sci Rep. 2025;15(1):7643. doi: 10.1038/s41598-025-91659-9 40038467 PMC11880298

[52] Hu W, Xiao Y, Feng M, Pu X, Shi W, Yang H, et al. Hydrogeochemical insights into the features, genesis and availability of groundwater quality in a densely agricultural plain on Yungui Plateau. Environ Earth Sci. 2024;83(22). doi: 10.1007/s12665-024-11892-9

[53] Xiao Y, Liu K, Hao Q, Xiao D, Zhu Y, Yin S, et al. Hydrogeochemical insights into the signatures, genesis and sustainable perspective of nitrate enriched groundwater in the piedmont of Hutuo watershed, China. CATENA. 2022;212:106020. doi: 10.1016/j.catena.2022.106020

[54] Zhang Y, Xiao Y, Yang H, Wang L, Wang J, Hu W, et al. Hydrogeochemical features, genesis, and quality appraisal of confined groundwater in a typical large sedimentary plain. Water Environment Research. 2024;96(8). doi: 10.1002/wer.11088 39091045

[55] Wang L, Xiao Y, Zhao Z, Qin G, Hu W, Chen H, et al. Hydrogeochemical heterogeneity, driving forces, and human health risk of groundwater in an arid alluvial fan plain on Tibetan Plateau. Human and Ecological Risk Assessment: An International Journal. 2025;31(3–4):412–33. doi: 10.1080/10807039.2025.2477503

[56] Hu W, Xiao Y, Wang L, Zhang Y, Feng M, Shi W, et al. Spatial variability, source identification, and partitioning of groundwater constituents in a typical lakeside plain on Yungui Plateau. Process Safety and Environmental Protection. 2024;191:2402–15. doi: 10.1016/j.psep.2024.09.107

[57] Rashid A, Khan S, Ayub M, Sardar T, Jehan S, Zahir S, et al. Mapping human health risk from exposure to potential toxic metal contamination in groundwater of Lower Dir, Pakistan: Application of multivariate and geographical information system. Chemosphere. 2019;225:785–95. doi: 10.1016/j.chemosphere.2019.03.066 30903852

[58] Tariq M, Rashid A, Khattak SA, Ali L, Shah MT. Hydrogeochemical characteristics, source distribution, and health risk of high-fluoride groundwater in Swabi, Pakistan. Environ Monit Assess. 2024;197(1):35. doi: 10.1007/s10661-024-13458-5 39641805

[59] Yin S, Xiao Y, Han P, Hao Q, Gu X, Men B, et al. Investigation of Groundwater Contamination and Health Implications in a Typical Semiarid Basin of North China. Water. 2020;12(4):1137. doi: 10.3390/w12041137

[60] Hao Q, Xiao Y, Chen K, Zhu Y, Li J. Comprehensive Understanding of Groundwater Geochemistry and Suitability for Sustainable Drinking Purposes in Confined Aquifers of the Wuyi Region, Central North China Plain. Water. 2020;12(11):3052. doi: 10.3390/w12113052

[61] WHO. Guidelines for drinking-water quality, 4th edition Incorporating the First Addendum. World Health Organization; 2017. 28759192

[62] GAQS. Standards for groundwater quality (GB/T 14848-2017). Beijing: General Administration of Quality Supervision; 2017.

[63] NHCPRC. National food safety standard — Drinking natural mineral water (GB 8537-2018). National Health Commission of the People’s Republic of China; 2018.

[64] Qu S, Wang J, Zhang K, Fan M, Zhao Y, Yang X, et al. Fate and risks of potentially toxic elements associated with lacustrine groundwater discharge: quantification, modeling, and biogeochemistry. Environ Int. 2025;202:109707. doi: 10.1016/j.envint.2025.109707 40737860

[65] Qu S, Zhao Y, Li M, Ren X, Wang C, Yang X, et al. Unveiling sources and fate of sulfate in lake-groundwater system combined Bayesian isotope mixing model with radon mass balance model. Water Res. 2025;282:123648. doi: 10.1016/j.watres.2025.123648 40252403

[66] Zhang Y, Xiao Y, Yang H, Wang S, Wang L, Qi Z, et al. Hydrogeochemical and isotopic insights into the genesis and mixing behaviors of geothermal water in a faults-controlled geothermal field on Tibetan Plateau. Journal of Cleaner Production. 2024;442:140980. doi: 10.1016/j.jclepro.2024.140980

[67] Rashid A, Khattak SA, Ali L, Zaib M, Jehan S, Ayub M, et al. Geochemical profile and source identification of surface and groundwater pollution of District Chitral, Northern Pakistan. Microchemical Journal. 2019;145:1058–65. doi: 10.1016/j.microc.2018.12.025

[68] Ayub M, Javed H, Rashid A, Khan WH, Javed A, Sardar T, et al. Hydrogeochemical properties, source provenance, distribution, and health risk of high fluoride groundwater: Geochemical control, and source apportionment. Environ Pollut. 2024;362:125000. doi: 10.1016/j.envpol.2024.125000 39313127

[69] Qu S, Ren X, Zhao Y, Mao H, Dong S, Yu R. Unveiling origin and enrichment of fluoride in the Daihai lake basin, China, using a hybrid hydrochemical and multi-isotopic method. Journal of Hydrology. 2025;656:133030. doi: 10.1016/j.jhydrol.2025.133030

[70] Peng C, He J-T, Wang M-L, Zhang Z-G, Wang L. Identifying and assessing human activity impacts on groundwater quality through hydrogeochemical anomalies and NO3-, NH4+, and COD contamination: a case study of the Liujiang River Basin, Hebei Province, P.R. China. Environ Sci Pollut Res Int. 2018;25(4):3539–56. doi: 10.1007/s11356-017-0497-x 29159440

[71] Xiao Y, Hao Q, Zhang Y, Zhu Y, Yin S, Qin L, et al. Investigating sources, driving forces and potential health risks of nitrate and fluoride in groundwater of a typical alluvial fan plain. Sci Total Environ. 2022;802:149909. doi: 10.1016/j.scitotenv.2021.149909 34525690

[72] Gibbs RJ. Mechanisms controlling world water chemistry. Science. 1970;170(3962):1088–90. doi: 10.1126/science.170.3962.1088 17777828

[73] Li K, Tian P, Cao Z, Qian H. Hydrochemical evolution of surface water and groundwater in Yiluo river basin, China and its formation mechanisms. Journal of Earth Sciences and Environment. 2025;47(3):537–54.

[74] Zhu C, et al. Hydrochemical characteristics and water quality dynamic analysis of shallow groundwater in Huaibei Plain. Hydrogeology & Engineering Geology. 2025;52(3):56–67. doi: 10.16030/j.cnki.issn.1000-3665.202409072

[75] Gaillardet J, Dupré B, Louvat P, Allègre CJ. Global silicate weathering and CO2 consumption rates deduced from the chemistry of large rivers. Chemical Geology. 1999;159(1–4):3–30. doi: 10.1016/s0009-2541(99)00031-5

[76] Alam SMK, Li P, Fida M. Groundwater Nitrate Pollution Due to Excessive Use of N-Fertilizers in Rural Areas of Bangladesh: Pollution Status, Health Risk, Source Contribution, and Future Impacts. Exposure and Health. 2024;16(1):159–82. doi: 10.1007/s12403-023-00545-0

[77] Ning H, Jiang W, Sheng Y, Wang K, Chen S, Zhang Z, et al. Comprehensive evaluation of nitrogen contamination in water ecosystems of the Miyun reservoir watershed, northern China: distribution, source apportionment and risk assessment. Environ Geochem Health. 2024;46(8):278. doi: 10.1007/s10653-024-02059-3 38958772

[78] Xie X, Wang Y, Ellis A, Su C, Li J, Li M, et al. Delineation of groundwater flow paths using hydrochemical and strontium isotope composition: A case study in high arsenic aquifer systems of the Datong basin, northern China. Journal of Hydrology. 2013;476:87–96. doi: 10.1016/j.jhydrol.2012.10.016

[79] Han G, Liu C-Q. Strontium isotope and major ion chemistry of the rainwaters from Guiyang, Guizhou Province, China. Sci Total Environ. 2006;364(1–3):165–74. doi: 10.1016/j.scitotenv.2005.06.025 16169575

[80] Wang Z-L, Zhang J, Liu C-Q. Strontium isotopic compositions of dissolved and suspended loads from the main channel of the Yangtze River. Chemosphere. 2007;69(7):1081–8. doi: 10.1016/j.chemosphere.2007.04.031 17531287

[81] Laudicina VA, Dazzi C, Delgado A, Barros H, Scalenghe R. Relief and calcium from gypsum as key factors for net inorganic carbon accumulation in soils of a semiarid Mediterranean environment. Geoderma. 2021;398:115115. doi: 10.1016/j.geoderma.2021.115115

[82] Chang C, Beckford HO, Ji H. Indication of Sr Isotopes on Weathering Process of Carbonate Rocks in Karst Area of Southwest China. Sustainability. 2022;14(8):4822. doi: 10.3390/su14084822

[83] de Caritat P, Kirste D, Carr G, McCulloch M. Groundwater in the Broken Hill region, Australia: recognising interaction with bedrock and mineralisation using S, Sr and Pb isotopes. Applied Geochemistry. 2005;20(4):767–87. doi: 10.1016/j.apgeochem.2004.11.003

[84] Cartwright I, Weaver T, Petrides B. Controls on 87Sr/86Sr ratios of groundwater in silicate-dominated aquifers: SE Murray Basin, Australia. Chemical Geology. 2007;246(1–2):107–23. doi: 10.1016/j.chemgeo.2007.09.006

[85] Miruo L, Yanzhong W, Yingchang C, Shuping W, Qiangwang X, Xiuyu D. Sources of Ca2+ in the major carbonate cements in Eocene sandstones and conglomerates: Evidence from Sr isotopes, Sr/Ca ratios, and rare-earth elements. Marine and Petroleum Geology. 2020;120:104568. doi: 10.1016/j.marpetgeo.2020.104568

[86] Rashid A, Ayub M, Khan S, Ullah Z, Ali L, Gao X, et al. Hydrogeochemical assessment of carcinogenic and non-carcinogenic health risks of potentially toxic elements in aquifers of the Hindukush ranges, Pakistan: insights from groundwater pollution indexing, GIS-based, and multivariate statistical approaches. Environ Sci Pollut Res Int. 2022;29(50):75744–68. doi: 10.1007/s11356-022-21172-3 35661301

