# Supplementary material for: Characterization of strontium-rich groundwater in a typical alpine basin on Tibetan Plateau: Implications for sustainable exploitation and development
Source: PLoS One. 2025 Sep 10;20(9):e0331449. doi: 10.1371/journal.pone.0331449 (PMC12422519; doi:10.1371/journal.pone.0331449)
Supplement: S1 Table — The exposure factors include Exposure Frequency (EF, d/y), Exposure Duration (ED, years), Ingestion Rate (IR, L/d), Body Weight (BW, kg), Skin Surface Area (SA, cm2), Averaging Time (AT, h), Exposure Time (T, h), Event Frequency (EV, day), Volumetric Conversion Factor (CF, L/cm3), and Dermal Permeability Coefficient (Kp, cm/h). Each exposure factor lists the values for four demographic groups (Infants, Children, Adult Females, and Adult Males). The corresponding oral reference doses (RfDoral, mg/(kg × day)) for the contaminants of concern (NO3-, NO2-, NH4+) are listed alongside. (DOCX) [file pone.0331449.s001.docx]

**Supplementary material**

**S1 Table.** The exposure parameters and RfD_oral_ used in human health risk assessment.

| **Exposure** |  | | **Value** | | | **Contaminant** | **RfD_oral_** |
| --- | --- | --- | --- | --- | --- | --- | --- |
| **Parameter** | **Infants** | **Children** | | **Adult Females** | **Adult Males** |  | (mg/(kg × day)) |
| EF (d/y) | 365^a^ | 365^a^ | | 365^a^ | 365^a^ | NO_3_^-^ | 1.6^b^ |
| ED (years) | 0.5 ^a^ | 16.5^a^ | | 30^a^ | 30^a^ | NO_2_^-^ | 0.1^b^ |
| IR (L/d) | 0.65^a^ | 1.5^a^ | | 2.5^a^ | 2.75^a^ | NH_4_^+^ | 0.97^b^ |
| BW (kg) | 9.27^a^ | 36.66^a^ | | 62.26^a^ | 72.42^a^ |  |  |
| SA (cm^2^) | 4200^a^ | 11400^a^ | | 16000^a^ | 17900^a^ |  |  |
| AT (h) | 183^a^ | 6023^a^ | | 10950 ^a^ | 10950^a^ |  |  |
| T (h) | 1^a^ | 1^a^ | | 1^a^ | 1^a^ |  |  |
| EV (day) | 1^a^ | 1^a^ | | 1^a^ | 1^a^ |  |  |
| CF (L/cm^3^) | 0.001^a^ | 0.001^a^ | | 0.001^a^ | 0.001^a^ |  |  |
| K_p_ (cm/h) | 0.001^a^ | 0.001^a^ | | 0.001^a^ | 0.001^a^ |  |  |

a represented that sourced from [Hu et al. (2024)](#_ENREF_2); b represented that sourced from [Askari et al. (2024)](#_ENREF_1).

References

Askari, M. et al., 2024. Bottled water safety evaluation: A comprehensive health risk assessment of oral exposure to heavy metals through deterministic and probabilistic approaches by Monte Carlo simulation. Food and Chemical Toxicology, 185: 114492. DOI:10.1016/j.fct.2024.114492

Hu, W. et al., 2024. Hydrogeochemical insights into the features, genesis and availability of groundwater quality in a densely agricultural plain on Yungui Plateau. Environmental Earth Sciences, 83(22): 628. DOI:10.1007/s12665-024-11892-9
